# Thoracic Empyema: A 12-Year Study from a UK Tertiary Cardiothoracic Referral Centre

**DOI:** 10.1371/journal.pone.0030074

**Published:** 2012-01-20

**Authors:** Daniel J. B. Marks, Marie D. Fisk, Chieh Y. Koo, Menelaos Pavlou, Lorraine Peck, Simon F. Lee, David Lawrence, M. Bruce Macrae, A. Peter R. Wilson, Jeremy S. Brown, Robert F. Miller, Alimuddin I. Zumla

**Affiliations:** 1 Department of Cardiology, The Heart Hospital, University College London Hospitals NHS Foundation Trust, London, United Kingdom; 2 Department of Medicine, University College London, London, United Kingdom; 3 Research Department of Infection and Population Health, University College London Medical School, University College London, London, United Kingdom; 4 Division of Infection and Immunity, Institute of Molecular and Cellular Biology, Porto, Portugal; 5 Department of Cardiothoracic Surgery, The Heart Hospital, University College London Hospitals NHS Foundation Trust, London, United Kingdom; 6 Department of Clinical Microbiology, University College London Hospitals NHS Foundation Trust, London, United Kingdom; 7 Centre for Respiratory Research, University College London, London, United Kingdom; 8 Department of Infection, University College London Medical School, University College London, London, United Kingdom; Institute of Infectious Diseases and Molecular Medicine, South Africa

## Abstract

**Background:**

Empyema is an increasingly frequent clinical problem worldwide, and has substantial morbidity and mortality. Our objectives were to identify the clinical, surgical and microbiological features, and management outcomes, of empyema.

**Methods:**

A retrospective observational study over 12 years (1999–2010) was carried out at The Heart Hospital, London, United Kingdom. Patients with empyema were identified by screening the hospital electronic ‘Clinical Data Repository’. Demographics, clinical and microbiological characteristics, underlying risk factors, peri-operative blood tests, treatment and outcomes were identified. Univariable and multivariable statistical analyses were performed.

**Results:**

Patients (n = 406) were predominantly male (74.1%); median age = 53 years (IQR = 37–69). Most empyema were community-acquired (87.4%) and right-sided (57.4%). Microbiological diagnosis was obtained in 229 (56.4%) patients, and included streptococci (16.3%), staphylococci (15.5%), Gram-negative organisms (8.9%), anaerobes (5.7%), pseudomonads (4.4%) and mycobacteria (9.1%); 8.4% were polymicrobial. Most (68%) cases were managed by open thoracotomy and decortication. Video-assisted thoracoscopic surgery (VATS) reduced hospitalisation from 10 to seven days (*P* = 0.0005). All-cause complication rate was 25.1%, and 28 day mortality 5.7%. Predictors of early mortality included: older age (*P* = 0.006), major co-morbidity (*P* = 0.01), malnutrition (*P* = 0.001), elevated red cell distribution width (RDW, P<0.001) and serum alkaline phosphatase (*P* = 0.004), and reduced serum albumin (*P* = 0.01) and haemoglobin (*P* = 0.04).

**Conclusions:**

Empyema remains an important cause of morbidity and hospital admissions. Microbiological diagnosis was only achieved in just over 50% of cases, and tuberculosis is a notable causative organism. Treatment of empyema with VATS may reduce duration of hospital stay. Raised RDW appears to associate with early mortality.

## Introduction

Thoracic empyema, defined as pus in the pleural cavity, is associated with considerable morbidity and mortality worldwide. A number of studies among adults and children consistently show that its incidence continues to increase in Western countries despite improvements in medical care and availability of effective antimicrobial therapy [1]–[8]. Thoracic empyema currently affects over 65,000 patients each year in the US and UK, at an estimated cost of $500 million to health services [9], [10]. Specific microbiological diagnosis is required to guide choice of antibiotic therapy, and is usually achieved through evaluation of pleural fluid/exudate and blood cultures. The causative microorganism is, however, only identified in approximately half of cases. A wide range of microbes have been isolated from empyema. Common bacterial pathogens include *Streptococcus milleri* group species, *Streptococcus pneumoniae*, methicillin-sensitive *Staphylococcus aureus* (MSSA) and the *Enterobacteriaceae* group [11], [12]. Worldwide, *Mycobacterium tuberculosis* (TB) is one of the most important causes of pleural infection, often associated with HIV co-infection [1], [13], [14]. In the United Kingdom, there is no routine screening for TB in patients presenting with empyema.

The treatment of empyema includes protracted courses of various single and multiple antibiotics. The majority require surgical drainage, which necessitates prolonged inpatient hospital admission and substantial costs for service providers [1], [5], [15], [16]. The overall case fatality remains high and there is a need to identify factors that could improve treatment outcomes. These include rapid identification of causative microorganisms and administration of pathogen-specific therapy; determination of optimal surgical interventions; and improved characterisation of risk factors, clinical features and biomarkers for detecting patients with poor prognosis. We aimed to identify the clinical characteristics, risk factors, microbial aetiology, operative management and outcomes, and predictors of early mortality of empyema cases presenting to a referral cardiothoracic centre in the UK, to provide informed guidance on developing improved management and cost-effective service delivery.

## Methods

**IRB/Ethics**: Data were analyzed anonymously, using publicly available secondary data, therefore no ethics statement is required for this work.

**Study design:** A retrospective study of all patients with empyema presenting over a period of 12 years (1999–2010).

### Study population, review of case notes and creation of study database

Patients were identified using a validated search strategy [17] by screening the hospital electronic Clinical Data Repository (CDR). The case definition of empyema was the presence of pus or other evidence of active infection in the pleural cavity [1], [9]. Inclusion criteria were: the presence of pleural fluid that was macroscopically purulent; pleural fluid with positive cultures or Gram/AAFB stain; pleural fluid pH<7.2 with clinical indicators of infection including fever, peripheral blood leukocytosis and/or elevated C-reactive protein (CRP); or histological confirmation of pus in the pleural cavity. Exclusion criteria were: alternative diagnosis on case note review; duplicate entry or re-admission for same empyema. The Heart Hospital only treats adult patients (>18 years).

The following information was obtained and recorded: patient data, medical and surgical history, clinical characteristics, presence of underlying risk factors, data from investigations (imaging, microbiology, histopathology of pleural tissue, biochemistry, serology, HIV testing, haematology, liver and renal function tests), medical and surgical treatment, antibiotic usage, and duration of inpatient admission. The outcomes of management were recorded, including all-cause mortality at 28 days and all-cause post-operative complication rate.

### Microbiology

Microbiological data recorded included results from pleural fluid, pleural tissue, blood and sputum cultures, specific mycobacterial and fungal cultures, molecular diagnostic tests and nasal carriage of methicillin-resistant *S. aureus* (MRSA). Tuberculous empyema was diagnosed only if *M. tuberculosis* bacilli were isolated from empyema pus and/or tissue. Post-operative wound infection was classified according to ASEPSIS [18] and CDC [19] criteria.

### Definitions of community-acquired versus hospital-acquired empyema

Infection status was defined as hospital-acquired if the onset of empyema or underlying pneumonia had occurred ≥two days after hospitalization, if the patient had been hospitalized within the preceding four weeks, or if it arose as a complication of an invasive thoracic procedure.

### Surgical procedures

Procedures used for surgical management of empyema were recorded.

### Statistical analyses

Comparisons were performed using the Mann-Whitney U test or one-way ANOVA with Bonferroni post-tests for continuous data, and Fisher's exact test or χ2 test for event frequencies as appropriate. Probability of 28 day mortality was modelled using logistic regression; in distributions demonstrating skew log_10_ values were used. Univariable and multivariable analyses were performed, although due to the low event frequency of mortality a multivariable model was fitted that was restricted to three factors (selected on the basis of significance from univariable analyses). Statistical analyses were conducted using GraphPad Prism v4.01 (GraphPad Software Inc., San Diego, USA) and STATA Version 10 (StataCorp LP, Texas, USA). A *P* value <0.05 was considered significant.

## Results

A total of 406 patients with empyema were identified from 526 initially selected by screening CDR. **Table 1** shows the microbiological and demographic characteristics of these empyema cases. Patients were predominantly male (74.1%, *P*<0.001, OR = 2.87, 95%CI = 2.13–3.85), with a median age of 53 years (IQR = 37–69). Empyema were principally community-acquired (87.4%, *P*<0.0001, OR = 6.96, 95%CI = 4.89–9.90) and right-sided (57.4%, *P* = 0.03, OR = 1.38, 95%CI = 1.04–1.82); four patients had bilateral empyema. The majority were loculated (59.6%, *P* = 0.007, OR = 1.48, 95%CI = 1.12–1.95).

**Table 1:** Microbiological characteristics of empyema.

		Age	Male	Hospital-acquired
		(median, IQR)		
All	(n = 406)	53 (37–69)	301 (74.1%)	51 (12.6%)***
Gram-Positive Cocci	(n = 142, 35.0%)			
Streptococci	(n = 79, 19.5%)			
*S. milleri*	(n = 17, 4.2%)	62 (41–73)	14 (82.4%)	3 (17.6%)
*S. pneumoniae*	(n = 39, 9.6%)	40 (35.5–64)	19 (48.7%)***	2 (5.1%)***
Other Streptococci	(n = 10, 2.5%)	37.5 (33–55.5)	6 (60.0%)	0 (0.0%)*
Enterococci	(n = 13, 3.2%)	46 (35–57)	10 (76.9%)	4 (30.8%)
Staphylococci	(n = 63, 15.5%)			
MSSA	(n = 36, 8.9%)	49 (33–70)	31 (86.1%)	8 (22.2%)*
MRSA	(n = 27, 6.7%)	60 (36.5–69)	23 (85.2%)	12 (44.4%)
Anaerobes	(n = 23, 5.7%)	62 (45.5–73)	17 (73.9%)	6 (26.1%)
Gram-Negative Bacilli	(n = 41, 10.1%)			
Enterobacteriaceae	(n = 23, 5.7%)	54 (36.5–72)	17 (73.9%)	9 (39.1%)
*P. aeruginosa*	(n = 18, 4.4%)	56.5 (43.5–67.25)	14 (77.8%)	5 (27.8%)
Other bacteria	(n = 13, 3.2%)	50 (41–63)	10 (76.9%)	3 (23.1%)
Mycobacteria	(n = 37, 9.1%)	38 (31–51)***	28 (75.7%)	1 (2.7%)***
Fungi	(n = 9, 2.2%)	50 (35–60)	7 (77.8%)	4 (44.4%)
Polymicrobial	(n = 34, 8.4%)	50 (33.5–69.5)	27 (79.4%)	13 (38.2%)
No Organism Identified	(n = 177, 44.0%)	56 (40–69)	135 (76.3%)	10 (5.6%)***

Data are n (%) unless otherwise stated. P values for hospital-acquired infection refer to significance in favour of community acquisition.

*P<0.05,

***P<0.001.

Positive cultures were obtained from 229 (56.4%) patients: from pleural fluid/pus in 174 (42.9%) and peripheral blood in 61 (15.0%). Direct microscopy of pleural fluid/pus for acid and alcohol fast bacilli was reported in 313 (77.1%) patients, mycobacterial cultures in 348 (85.7%) and histology of the pleural specimen in 316 (77.8%). Specific fungal cultures were performed in 148 (36.5%) patients, and serum cryptococcal antigen tested in five.

**Figure 1:**
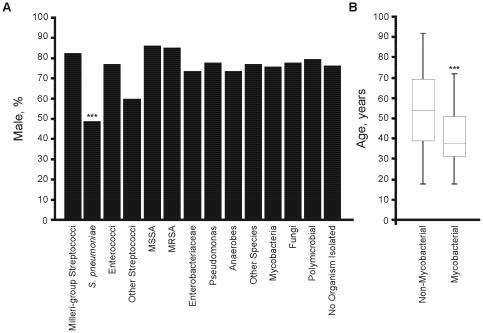
Microbiological and demographic characteristics. (A) Variation in gender across microbiological subgroups. (B) Patients with mycobacterial infections were significantly younger; data shown as median, IQR (boxes) and total range. EC, enterococci; EB, Enterobacteriaceae; Ps, Pseudomonads. ***P<0.001.

Organisms identified included streptococci (16.3%), staphylococci (15.5%), Gram-negative organisms (8.9%), anaerobes (5.7%), pseudomonads (4.4%) and mycobacteria (9.1%); 34 cultures (8.4%) were polymicrobial (**Table 1**). There was evidence of gender difference across the microbiological subgroups (χ2 = 19.12, *P* = 0.01), with male predominance in all except *S. pneumoniae* (**Figure 1A**). Patients with mycobacterial infection were significantly younger (median 38 years, IQR 31–51, *P*<0.001) (**Figure 1B**), but no other age differences were observed. *S. pneumoniae* (*P*<0.0001, OR = 18.5, 95%CI = 3.92–87.4), *S. milleri* group (*P* = 0.03, OR = 21.0, 95%CI = 0.97–454.3), MSSA (*P* = 0.03, OR = 3.50, 95%CI = 1.26–9.73), *M. tuberculosis* (*P*<0.0001, OR = 36.00, 95%CI = 4.44–291.7), and empyema with no organism isolated (*P*<0.0001, OR = 16.70, 95%CI = 8.27–33.74) were principally community-acquired; whereas MRSA, *Enterobacteriaceae*, anaerobes, *Pseudomonas* species and polymicrobial infections were commoner in the hospital-acquired group. There was no difference between the groups in lateralisation of empyema but there was evidence of variation in loculation (χ2 = 20.05, *P* = 0.01), which was more frequent with *S. pneumoniae* (*P* = 0.02, OR = 3.33, 95%CI = 1.25–8.88).

The distribution of risk factors among the 406 patients with empyema and evidence of their variation between microbiological groups are shown in **Table 2**. These included thoracic surgery and trauma; previous empyema; malnutrition; active sepsis; diabetes mellitus; use of steroids; homelessness; intravenous drug misuse; and HIV infection. Individual categories achieving significance included over-representation of Gram-negative and anaerobic species in patients with a past history of empyema (*P* = 0.03, OR = 3.87, 95%CI = 1.18–12.68); and enterococci (*P* = 0.05, OR = 7.35, 95%CI = 1.31–43.05), *Enterobacteriaceae* (*P* = 0.04, OR = 4.29, 95%CI = 1.25–14.74), *Pseudomonas* species (*P* = 0.04, OR = 5.20, 95%CI = 1.25–21.58) and polymicrobial infection (*P* = 0.007, OR = 4.40, 95%CI = 1.59–12.20) in septic patients. Characteristics of individuals with HIV co-infection are shown in **Table 3**; in this group the median CD4 count was 380 cells/µL.

**Table 2:** Distribution of risk factors and evidence of variation between microbiological groups.

Risk Factor	Number of Patients	χ2	P value
Thoracic Surgery	73 (18.0%)	26.46	0.009
Thoracic Trauma	27 (6.7%)	23.19	0.003
Previous empyema	29 (7.1%)	28.21	0.005
Malnourished	188 (46.3%)	41.47	<0.0001
Septic	139 (34.2%)	84.32	<0.0001
Smoker	118 (29.1%)	15.65	0.21
COPD	25 (6.2%)	16.12	0.19
Diabetes Mellitus	33 (8.1%)	25.74	0.01
Malignancy	35 (8.6%)	11.24	0.51
Chemotherapy	17 (4.2%)	15.02	0.24
Thoracic radiotherapy	5 (1.2%)	14.39	0.28
Steroids	50 (12.3%)	34.22	0.0006
Immunosuppressant	32 (7.9%)	11.27	0.51
Homeless	19 (4.7%)	25.3	0.01
Alcohol misuse	60 (14.8%)	18.38	0.10
Intravenous drug user	36 (8.9%)	28.38	0.005
HIV-infected	14 (3.4%)	27.18	0.007
Data are n (%).			

P values refer to comparisons between the frequencies of microbial aetiologies in patients with the risk factor and those in whom it was not present.

**Table 3:** Characteristics of patients with HIV infection.

Patient	CD4	Viral load	Anti-retroviral therapy	Organism
	(cells/µL)	(copies/mL)		
1	50	>500,000	No	*S. pneumoniae*
2	340	100	Yes	*S. pneumoniae*
3	Not done	Not done	No	*S. pneumoniae*
4	630	37,000	No	*S. pneumoniae*
5	430	59,000	No	*S. pneumoniae*
6	890	<50	No	*M. tuberculosis*
7	180	<50	Yes	*M. tuberculosis*
8	640	38,000	No	*M. tuberculosis*
9	68	Not done	No	MSSA
10	Not done	Not done	Yes	*H. influenzae*
11	570	9,000	Yes	Candida species
12	30	14,000	Yes	Polymicrobial (milleri-group streptococci, *P. mirabilis*, *C. albicans*, *A. fumigatus*)
13	420	<50	Yes	None isolated
14	323	<50	Yes	None isolated

**Figure 2:**
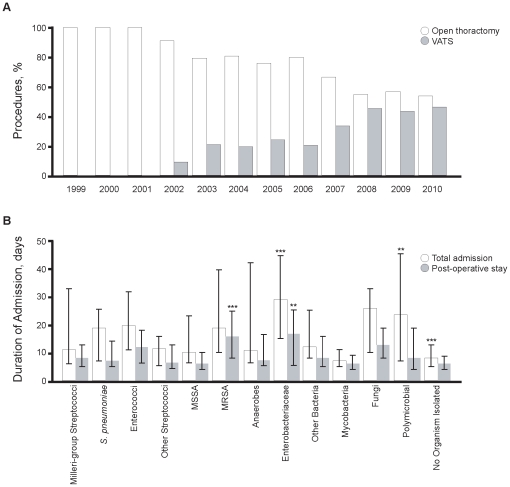
Operative management. (A) There was a clear trend to increased use of VATS over the course of the study (*P*<0.001). (B) Total and post-operative durations of admission varied across the microbiological subgroups. **P<0.01, ***P<0.001.

The majority of patients were managed by open thoracotomy and decortication (n = 277, 68.2%). Video-assisted thoracoscopic surgery (VATS) was used in 116 (28.6%) patients over the study period; with 17 (14.7%) converted to open procedures. There was a significant trend towards an increasing use of VATS over time (χ2 = 41.85, *P*<0.0001; **Figure 2A**). The median duration of total hospital admission was nine (6–19) days, and post-procedure admission was seven (4.68–13) days. Duration of hospital admission stay was shorter with VATS with a median of seven (5–13) days in total (*P*<0.0001) and five days post-procedure (4–8.5) days (*P*<0.0001); the corresponding durations for open thoracotomy were 10 (6.5–21) days and seven (5–14) days respectively. This was reflected in the trend to briefer admissions over the course of the study, with median durations of 26 (15–40.5) days in total and 16.5 (9–21) days post-procedure in 1999 falling to six (4.5–15.5, *P* = 0.0005) and five (4–9, *P* = 0.003) days respectively in 2010. Admission length also varied across microbiological group (*P*<0.0001; **Figure 2B**), and between community and hospital acquired empyema. Total duration was protracted in patients with *Enterobacteriaceae* (*P*<0.001) and polymicrobial infections (*P* = 0.006), and shorter in those in whom no organism was isolated (*P*<0.0001). Post-operative admission was prolonged in patients with MRSA (*P*<0.0001) or *Enterobacteriaceae* (*P* = 0.007) infections. Nosocomial infections were associated with significantly longer total and post-operative admissions with medians of 17 (8–35.5, *P* = 0.0006) and eight (5–21, *P* = 0.04) days respectively, compared to nine (6–17) and six (5–12) days for those acquired in the community.

The all-cause complication rate was 25.1%, the most frequent being wound infection. These occurred in 23 (5.7%) patients by CDC criteria [18], [19], in whom 18 were superficial and five were deep infections, with no significant difference between the open thoracotomy and VATS groups. Other complications included unsuccessful procedure/incomplete drainage (4.9%), pneumothorax (3.4%), intrathoracic haemorrhage (3.2%), systemic sepsis (1.5%), lower respiratory tract infection, cardiac dysrhythmia, infection at other sites, gastrointestinal haemorrhage, and acute kidney injury. Patients with complications had prolonged hospital admissions, with medians of 16 (10–32, *P*<0.0001) total and 13 (7–22.5, *P*<0.0001) post-operative days compared with eight (5–16) and six (4–9) days in those who had an uncomplicated recovery. The 28 day mortality was 5.7%, with no significant differences across the microbiological groups, or between open thoracotomy and VATS. Associations of mortality with clinical characteristics and blood test variables were evaluated by univariable and multivariable analyses (**Table 4**). In univariable analysis patient age (*P* = 0.006), malnutrition (*P* = 0.001) and co-morbidity (*P* = 0.01) were predictive of mortality, as were low pre-operative serum albumin (*P* = 0.01) and haemoglobin (*P* = 0.04), and high serum alkaline phosphatase (*P* = 0.004) and red cell distribution width (RDW, *P*<0.001); haemoglobin and RDW values were not independent. Other variables including white cell count, CRP (on admission, or peak value), platelet count and renal function were not associated with mortality. A p value of 0.1 was used as the threshold for initial selection of factors considered for multivariable analysis. Subsequently, given the small number of events (deaths) among this patient group, a model was developed limited to three predictors, with age, RDW and alkaline phosphatase selected for inclusion. A forward selection stepwise procedure for the multivariable model showed association of mortality with RDW (*P* = 0.001, OR = 1.36 per 1 unit increase, 95%CI = 1.14–1.63) and alkaline phosphatase (*P* = 0.03, OR = 2.63 per 1 unit increase in the log_10_ value, 95%CI = 1.08–6.44).

**Table 4:** Association between clinical characteristics and blood tests, and mortality.

Variable	Survivors	Died	Odds ratio	p	Missing
			(95% CI)		values
***Clinical Characteristics***					
Age (years)	52 (36–68)	65 (52–73)	1.44^1^ (1.11–1.87)	0.006	0
Male gender	286 (74.7%)	15 (65.2%)	0.64 (0.26–1.55)	0.318	0
Smoker	110 (35.7%)	8 (40.0%)	1.2 (0.48–302.00)	0.699	78
Malnourished	166 (43.6%)	22 (95.6%)	28.5 (3.80–213.50)	0.001	2
Co-morbidities	76 (19.8%)	10 (43.5%)	3.11 (1.31–7.36)	0.01	0
***Blood tests***					
Alanine transaminase (IU/L)	25 (16–44)	35 (18–59)	1.33^2^ (0.74–2.42)	0.342	99
Albumin (g/L)	32 (27–38)	27 (24–32)	0.92 (0.86–0.98)	0.014	89
Alkaline phosphatase (IU/L)	114 (86–172)	151 (125–262)	3.41^2^ (1.48–7.84)	0.004	91
APTT (sec)	35 (32–40)	37 (34–40)	1.02 (0.97–1.06)	0.452	85
Bilirubin (µmol/L)	7 (5–10)	7 (4–13)	1.10^2^ (0.48–2.51)	0.823	96
Creatinine (µmol/L)	68 (56–84)	69 (52–99)	1.56^2^ (0.57–4.24)	0.387	58
CRP, admission (mg/L)	100 (34–206)	87 (44–208)	0.99^3^ (0.96–1.04)	0.960	48
CRP, peak (mg/L)	225 (132–301)	205 (136–312)	1.00^3^ (0.96–1.04)	0.954	41
Haemoglobin (g/dL)	11.1 (9.9–12.4)	10.5 (9.4–11.3)	0.76 (0.59–0.98)	0.038	10
INR	1.04 (0.99–1.12)	1.04 (0.98–1.17)	1.01^4^ (0.81–1.27)	0.917	75
Mean platelet volume (fL)	9.5 (8.9–10.1)	9.9 (9.1–10.0)	1.00^4^ (0.96–1.05)	0.822	59
Neutrophils (×10^9^/L)	7.0 (5.0–10.3)	7.3 (5.4–10.7)	1.27^2^ (0.56–2.86)	0.563	59
Platelets (×10^9^/L)	430 (314–569)	324 (210–525)	0.76^5^ (0.58–1.00)	0.053	58
PTT (sec)	11.2 (10.7–12.0)	11.6 (10.5–12.3)	1.01 (0.87–1.16)	0.937	72
Red cell distribution width (%)	14.7 (13.7–16.1)	17.1 (15.5–19.4)	1.36 (1.17–1.59)	<0.001	59
Urea (mmol/L)	4.2 (3.3–6.0)	6.8 (3.6–9.5)	2.15^2^ (0.98–4.71)	0.055	59
White cell count (×10^9^/L)	10.2 (7.7–13.6)	10.2 (8.2–13.3)	1.10^2^ (0.39–3.11)	0.85	58

Data shown as n (%) or median (IQR). Odds ratios:

1 per 10 years,

2 per log10 units,

3 per 10 mg/L,

4 per 0.1 units,

5 per 100×10^9^/L).

## Discussion

This is to date the largest single centre series on empyema described, and shows that empyema remains an important cause of morbidity, mortality and hospital admissions in the UK. The results highlight several important points. First, a microbiological diagnosis was only achieved in just over 50% of 406 patients studied, highlighting the necessity for development and evaluation of more sensitive and rapid diagnostics for early identification of the specific microbial aetiologies. This would guide early targeted antimicrobial therapy and likely influence clinical outcomes. Second, it is important to note that is TB is important cause of empyema and may be easily overlooked: 37 cases were identified where biological specimens were sent with specific requests for mycobacterial investigation. Only 313 out of 406 cases of empyema had mycobacterial staining requested, even though this hospital has high awareness of TB as a differential diagnosis. Since the incidence of TB in the UK and elsewhere in Europe is increasing [13], it now becomes imperative that all patients with empyema are routinely screened. Third, use of VATS led to reduced median duration of hospital stay and may therefore reduce costs of empyema treatment; its cost-effectiveness should now be evaluated against other medical and surgical treatments. Finally, raised RDW appeared to be strongly associated with early mortality. This requires further prospective evaluation as a biomarker for identifying patients at high risk of early death. Adoption of all these points would be predicted to lead to shorter inpatient admissions, improved treatment outcomes and reduced costs of care in an increasingly resource constrained health service setting.

Comparison of our study with the UK MIST-1 trial [20] and a Danish multicentre descriptive series [12] revealed several commonalities but also important differences. The median age in our cohort was younger (10 and seven years respectively) and rates of co-morbidity were correspondingly lower; this may partially reflect referral bias to our tertiary cardiothoracic centre for operative management. Our study confirmed a right-sided predominance of empyema at an approximate ratio of 1.2∶1 [9], which closely follows the normal right∶left lung volume ratios [21]. The distribution of causative bacterial organisms identified was similar to recent series [12], [20], except that by contrast with MIST-1 we observed a lower frequency of milleri-group streptococci and a corresponding increase in *S. pneumoniae*.

Male predominance was observed in all microbiological categories except *S. pneumoniae*, consistent with its established epidemiology [22]. The role of male gender as a risk factor for immunocompromise and trauma may partially explain the predisposition to staphylococcal, Gram-negative, anaerobic and mycobacterial infections. The microbiology of nosocomial infections corresponded with that of other series, in which *Klebsiella* species and other Gram-negative organisms, and *Pseudomonas* species, were more common in patients with chronic disease requiring frequent hospitalization [23] and intensive care [24]. Sepsis was predictably more common in Gram-negative and polymicrobial infections, and less frequent in patients with tuberculosis or among those in whom no organism could be isolated, presumably since bacterial loads in the pleural fluid are likely to be lower with reduced rates of bacteraemia. Patients with HIV co-infection comprised an important subgroup (3.4%), despite an earlier contradictory report [25]. The predominance of *S. pneumoniae* and *M. tuberculosis* infections follows the patterns seen in pneumonia among HIV-infected individuals. The absence of a clear association with CD4 count is consistent with another series [26] and might reflect the effects of HIV on innate immunity beyond those on lymphocyte function [27].

Molecular assays, were only available in the latter part of this study, however a significant proportion of new diagnoses were established with their use. Several series illustrate that using assays based on 16 S RNA PCR increases the bacteriological diagnostic yield in empyema by 17–49% [9], [28] and concerns that the infective/inflammatory biochemical milieu in empyema might contain molecular inhibitors of PCR reactions do not appear substantiated [29]. Our study also highlighted a high rate of tuberculous empyema cases; *M. tuberculosis* was the second most commonly isolated single pathogen after *S. pneumoniae*. This reflects a combination of a high degree of clinical awareness at our hospital, and patient demographics in our catchment area. Patients in our study were referred for surgical drainage of pus, removal of empyema tissue or other intervention, and so uncomplicated TB pleural disease or tuberculous pulmonary parenchymal disease with pleural effusions were not included. Tuberculous effusions and empyema represent increasingly common presentations of mycobacterial disease in countries of high incidence, accounting for 4–10% of cases [14], particularly in the context of HIV co-infection [1]. Direct microscopy and culture of pleural fluid have diagnostic yields of 10–20% and 25–50%, respectively [30]. Additionally, pleural biopsy increases diagnostic yield to approximately 90% [31]. The introduction of rapid molecular assays for detection of *M. tuberculosis* DNA in sputum specimens should be evaluated on empyema pus and tissue and should be included in management guidelines if their utility is confirmed [32].

Since its routine introduction in 2002 in our hospital, we observed a clear trend over time towards increased use of VATS, which accounted for almost 50% of operations in the final year analysed. Thoracoscopic surgery is more likely to be successful with earlier referrals, before empyema enters the organising phase. Concordant with other studies suggesting that outcomes of minimally invasive surgery are superior to open procedures [5], [33]–[35], we found VATS led to shorter median durations of admissions, but no reduction in overall complication rate or mortality. We found no evidence of diminished wound infection rates with VATS in this cohort. This may reflect both an element of prophylaxis afforded by long-term antibiotic treatment of the underlying pleural infection (as these would usually possess good Gram-positive cover), and possibly less active monitoring related to shorter inpatient hospital stays.

Mortality in our study was low compared to most other series, in which rates range from 18–60% [3], [5], [36], potentially reflecting a combination of patient selection as well as operative experience. Nonetheless, we were able to identify several clinical variables that were associated with early post-operative death. These included low pre-operative haemoglobin, high RDW, low albumin and high alkaline phosphatase. The association with RDW probably does not merely reflect dimorphism attendant on the anaemia of chronic disease, since RDW was a stronger predicator of mortality than haemoglobin. Elevated RDW has previously been highlighted as an indicator of inflammatory stress, secondary to impaired iron mobilisation and availability [37], and correlates with disease activity and/or poor outcome in several chronic conditions including rheumatoid arthritis [38], inflammatory bowel disease [39], heart failure [40], and coronary artery disease [41]. A low albumin may be associated with mortality both as a marker of malnutrition and, along with elevated alkaline phosphatase, severe sepsis. Haemoglobin may assume additional importance as it is a risk factor amenable to correction with blood transfusion, which should be evaluated prospectively. Of interest, neither white cell count nor CRP predicted poor outcome, suggesting that while these variables have utility for monitoring disease activity they do not identify high risk patients.

The principal limitation of our study was its retrospective design and selected population, which as such has an implicit degree of selection bias. In addition, molecular diagnostics were introduced into practice later and not systematically used. Nonetheless, our search strategy has previously been formally validated (with a reported positive predictive value of 90.6%) [17] and these patients comprise the largest single centre cohort reported to date.

In conclusion, our study confirms and builds upon the microbiological findings of previous studies, and demonstrates that since its introduction use of VATS has been associated with shorter hospital admissions. Furthermore, we identified a number of prognostic factors predictive of early mortality, including the novel association with RDW. These should be investigated further for development of more accurate diagnostic algorithms and guidelines for management of empyema, which in turn could improve outcomes and reduce healthcare costs.

## References

[1] Lee SF, Lawrence D, Booth H, Morris-Jones S, Macrae B (2010). Thoracic empyema: current opinions in medical and surgical management.. Curr Opin Pulm Med.

[2] Christopoulou-Aletra H, Papavramidou N (2008). “Empyemas” of the thoracic cavity in the Hippocratic Corpus.. Ann Thorac Surg.

[3] Chen W, Lin YC, Liang SJ, Tu CY, Chen HJ (2009). Hospital-acquired thoracic empyema in adults: a 5-year study.. South Med J.

[4] Liang SJ, Chen W, Lin YC, Tu CY, Chen HJ (2007). Community-acquired thoracic empyema in young adults.. South Med J.

[5] Ferguson AD, Prescott RJ, Selkon JB, Watson D, Swinburn CR (1996). The clinical course and management of thoracic empyema.. QJM.

[6] Roxburgh CS, Youngson GG, Townend JA, Turner SW (2008). Trends in pneumonia and empyema in Scottish children in the past 25 years.. Arch Dis Child.

[7] Finley C, Clifton J, Fitzgerald JM, Yee J (2008). Empyema: an increasing concern in Canada.. Can Respir J.

[8] Li ST, Tancredi DJ (2010). Empyema hospitalizations increased in US children despite pneumococcal conjugate vaccine.. Pediatrics.

[9] Maskell NA, Batt S, Hedley EL, Davies CW, Gillespie SH (2006). The bacteriology of pleural infection by genetic and standard methods and its mortality significance.. Am J Respir Crit Care Med.

[10] Ahmed RA, Marrie TJ, Huang JQ (2006). Thoracic empyema in patients with community-acquired pneumonia.. Am J Med.

[11] Foster S, Maskell N (2007). Bacteriology of complicated parapneumonic effusions.. Curr Opin Pulm Med.

[12] Meyer CN, Rosenlund S, Nielsen J, Friis-Moller A (2011). Bacteriological aetiology and antimicrobial treatment of pleural empyema.. Scand J Infect Dis.

[13] Zumla A, Mwaba P, Huggett J, Kapata N, Chanda D (2009). Reflections on the white plague.. Lancet Infect Dis.

[14] Porcel JM (2009). Tuberculous pleural effusion.. Lung.

[15] Davies CW, Kearney SE, Gleeson FV, Davies RJ (1999). Predictors of outcome and long-term survival in patients with pleural infection.. Am J Respir Crit Care Med.

[16] Davies HE, Davies RJ, Davies CW (2010). Management of pleural infection in adults: British Thoracic Society Pleural Disease Guideline.. Thorax.

[17] Sogaard M, Kornum JB, Schonheyder HC, Thomsen RW (2011). Positive predictive value of the ICD-10 hospital diagnosis of pleural empyema in the Danish National Registry of Patients.. Clin Epidemiol.

[18] Wilson AP, Gibbons C, Reeves BC, Hodgson B, Liu M (2004). Surgical wound infection as a performance indicator: agreement of common definitions of wound infection in 4773 patients.. BMJ.

[19] Horan TC, Gaynes RP, Martone WJ, Jarvis WR, Emori TG (1992). CDC definitions of nosocomial surgical site infections -a modification of CDC definitions of surgical wound infections.. Am J Infect Control.

[20] Maskell NA, Davies CW, Nunn AJ, Hedley EL, Gleeson FV (2005). U.K. Controlled trial of intrapleural streptokinase for pleural infection.. N Engl J Med.

[21] Plathow C, Schoebinger M, Fink C, Ley S, Puderbach M (2005). Evaluation of lung volumetry using dynamic three-dimensional magnetic resonance imaging.. Invest Radiol.

[22] Robinson KA, Baughman W, Rothrock G, Barrett NL, Pass M (2001). Epidemiology of invasive Streptococcus pneumoniae infections in the United States, 1995–1998: Opportunities for prevention in the conjugate vaccine era.. JAMA.

[23] Chen CH, Hsu WH, Chen HJ, Chen W, Shih CM (2007). Different bacteriology and prognosis of thoracic empyemas between patients with chronic and end-stage renal disease.. Chest.

[24] Tu CY, Hsu WH, Hsia TC, Chen HJ, Chiu KL (2006). The changing pathogens of complicated parapneumonic effusions or empyemas in a medical intensive care unit.. Intensive Care Med.

[25] Coker RJ (1994). Empyema thoracis in AIDS.. J R Soc Med.

[26] Khwaja S, Rosenbaum DH, Paul MC, Bhojani RA, Estrera AS (2005). Surgical treatment of thoracic empyema in HIV-infected patients: severity and treatment modality is associated with CD4 count status.. Chest.

[27] Noursadeghi M, Katz DR, Miller RF (2006). HIV-1 infection of mononuclear phagocytic cells: the case for bacterial innate immune deficiency in AIDS.. Lancet Infect Dis.

[28] Blaschke AJ, Heyrend C, Byington CL, Obando I, Vazquez-Barba I (2011). Molecular analysis improves pathogen identification and epidemiologic study of pediatric parapneumonic empyema.. Pediatr Infect Dis J.

[29] Wrightson JM, Rahman NM, Novak T, Huggett JF, Maskell NA (2011). Pneumocystis jirovecii in pleural infection: a nucleic acid amplification study.. Thorax.

[30] Ferrer J (1997). Pleural tuberculosis.. Eur Respir J.

[31] Gopi A, Madhavan SM, Sharma SK, Sahn SA (2007). Diagnosis and treatment of tuberculous pleural effusion in 2006.. Chest.

[32] Boehme CC, Nabeta P, Hillemann D, Nicol MP, Shenai S (2010). Rapid molecular detection of tuberculosis and rifampin resistance.. N Engl J Med.

[33] Cardillo G, Carleo F, Carbone L, Di MM, Salvadori L (2010). Chronic postpneumonic pleural empyema: comparative merits of thoracoscopic versus open decortication.. Eur J Cardiothorac Surg.

[34] St Peter SD, Tsao K, Spilde TL, Keckler SJ, Harrison C (2009). Thoracoscopic decortication vs tube thoracostomy with fibrinolysis for empyema in children: a prospective, randomized trial.. J Pediatr Surg.

[35] Tong BC, Hanna J, Toloza EM, Onaitis MW, D'Amico TA (2010). Outcomes of video-assisted thoracoscopic decortication.. Ann Thorac Surg.

[36] Bender JM, Ampofo K, Sheng X, Pavia AT, Cannon-Albright L (2009). Parapneumonic empyema deaths during past century, Utah.. Emerg Infect Dis.

[37] Lippi G, Targher G, Montagnana M, Salvagno GL, Zoppini G (2009). Relation between red blood cell distribution width and inflammatory biomarkers in a large cohort of unselected outpatients.. Arch Pathol Lab Med.

[38] Baynes RD, Bothwell TH, Bezwoda WR, Gear AJ, Atkinson P (1987). Hematologic and iron-related measurements in rheumatoid arthritis.. Am J Clin Pathol.

[39] Cakal B, Akoz AG, Ustundag Y, Yalinkilic M, Ulker A (2009). Red cell distribution width for assessment of activity of inflammatory bowel disease.. Dig Dis Sci.

[40] van Kimmenade RR, Mohammed AA, Uthamalingam S, van der MP, Felker GM (2010). Red blood cell distribution width and 1-year mortality in acute heart failure.. Eur J Heart Fail.

[41] Isik T, Uyarel H, Tanboga IH, Kurt M, Ekinci M (2011). Relation of red cell distribution width with the presence, severity, and complexity of coronary artery disease.. Coron Artery Dis.

